# Social and Logistical Barriers to the Use of Reversible Contraception among Women in a Rural Indian Village

**Published:** 2008-06

**Authors:** Mary Ann Kirkconnell Hall, Rob B. Stephenson, Sanjay Juvekar

**Affiliations:** ^1^Hubert Department of Global Health, Rollins School of Public Health, Emory University, Atlanta, GA, USA; ^2^King Edward Memorial Hospital, Pune, Maharashtra, India

**Keywords:** Beliefs, Contraception, Contraception, Reversible, Contraceptive methods, Family planning, Women, India

## Abstract

Women in a small coastal village in western India were asked to explain their preference for female sterili-zation over modern reversible contraceptive methods. Married women aged 19+ years were interviewed in six focus groups (n=60) and individually (n=15) regarding contraceptive methods and their use and side-effects. Women publicly denied contraceptive use but privately acknowledged limited use. They obtained contraceptive information from other village women and believed that modern reversible methods and vasectomy have high physical and social risks, and fertility goals could be achieved without their use. Women felt that reversible contraception is undesirable, socially unacceptable, and usually unnecessary, although the achievement of fertility goals is likely due to the use of female sterilization with abortion as a back-up method. Economic migration of village men may also play a role. Although women with high social capital can effectively disseminate correct knowledge, the impact on the uptake of reversible method is uncertain.

## INTRODUCTION

Female sterilization is the most prevalent form of contraception in India, accounting for 76% of all use among women (1). Use rates of temporary modern contraceptive methods are very low. Previous studies have identified barriers to contraceptive use, which include the monetary and time costs of obtaining contraception (2,3), the social stigma of using contraceptives in an unsupportive setting (4,5), lack of knowledge (6,7), desire for more children (8-10), the costs of acquiring additional information (11), and worry over possible side-effects and fears that reversible methods are ineffective (4,7,8,12). Historically, Indian health providers have emphasized female sterilization; this may also limit the uptake of reversible contraception (8,12,13).

This paper explores beliefs of women regarding reversible contraception in a context where women are able to achieve their fertility goals using sterili-zation and periodic abstinence with abortion as a back-up method (14). The study was conducted in a rural Indian village where stigma regarding sexual immoderation and myths and misconceptions regarding side-effects of reversible contraception and vasectomy also contribute to the conviction of women that sterilization is superior. Unlike many other rural areas, the study area women had access to modern methods from a health clinic, a hospital, and pharmacies nearby. A greater understanding of the contraceptive decision-making process has the potential to inform programmes that seek to increase the uptake of temporary contraceptive methods in rural India.

An estimated 75% of all contraceptive users (84% of those who use a modern contraceptive method) in India rely on female sterilization, while vasectomy is used by just 2% of contracepting couples (10). For many women, female sterilization is the first and the only contraceptive method they use (15). The median age at sterilization is 25.7 years; this, however, varies from a low of 23.6 years in Andhra Pradesh in southern India to 30.5 years in Manipur in eastern India (16). In 2001, an estimated 15.8% of currently-married Indian women of reproductive age had an unmet need for contraception, i.e. they desired to space or limit pregnancies but were not using contraceptive methods to do so (7). The contraceptive prevalence rate of reversible methods has remained low; while supplies of reversible contraceptives are available free of charge to women in some areas, demand has been weak (12). In Maharashtra, although 60.9% of women are using contraception, only 7.6% are using reversible methods for spacing (17). Birth intervals of <36 months, which have been linked to increased maternal and child morbidity and mortality (18-22), are more common among women who do not use spacing methods. However, provision of free contraception and monetary incentives for their use had limited success in India (23).

## MATERIALS AND METHODS

The study village (population ~2000) is located on the Konkan Coast of Maharashtra state, India, and is sub-divided into smaller hamlets divided along family and caste lines. Two of the hamlets are reachable by *kanccha* (paved) road, and the rest are accessible by packed earth roads. No public transport is available in the village. Each hamlet has a nursery school for young children. The village has a primary school for children, but the nearest high school is in a town seven km distant.

Virtually, all adult men migrate to Pune and Mumbai for work; those who remain engage themselves in farming. Oral contraceptive pills, intrauterine devices (IUDs), and condoms are available from a nearby (3 km) health post, pharmacies in the nearest town (7 km), and the *taluka* (district) capital (20 km), although access to these sources may be limited by the lack of public transport. Sterilization procedures for both men and women and abortion services are available only at the hospital in the *taluka* capital.

All the ever-married village women were informed of the study in the spring of 2005 during a village-level household census conducted by a local non-governmental organization. Those who were interested attended a public meeting explaining the objectives of the study where they could volunteer to participate.

Each focus group consisted of 8–14 women, aged 19–50 years, from a single hamlet. The initial topic guide was constructed following a literature review and pilot tested in a neighbouring village. The focus-group discussion guide included questions on what women perceived to be overall contraceptive-use patterns in the village (although women were asked directly about the use of their own methods in interviews, we chose to ask the focus group general questions and allow women to share their own experiences if they wished), individual and community perceptions of different methods and their side-effects, and ideal and actual fertility preferences. Data from the focus groups were summarized in the field to refine the in-depth interview question guide, as the individual interviews took place after the focus groups had all been conducted.

Semi-structured in-depth interviews were conducted with currently-married women aged 25–53 years using a 27-item question guide, with questions regarding ideal and actual birth intervals, women's own use of contraceptives, and their knowledge of and access to modern contraceptives. The women who were interviewed individually had not participated in the focus groups.

Interview candidates were gathered using a ‘snowball' sampling technique, whereby the local research assistant recruited several women of varying caste and socioeconomic levels who indicated interest in participating in the study during the earlier census and public meeting. These women were then, in turn, asked to recruit other village women. Although such sampling is non-random and has the potential to introduce bias as subjects tend to identify other potential subjects by their mutually identifying characteristics, it allowed the researchers unparalleled access into the homes and lives of women of varying social status, literacy, age, and parity by establishing credibility and trust among research subjects. All focus groups and interviews were conducted in Marathi and were tape-recorded. After the completion of data collection, all interviews and discussions of focus groups were translated and transcribed into English from Marathi for analysis with VERBI Software's MAX Qualitative Data Analysis 2 (MAXqda2) program.

## RESULTS

### Family-planning use

Women in the focus groups were initially reluctant to speak about contraceptive use, even in the abstract, and several women in each group stated that a few village women use any methods other than periodic abstinence and female sterilization. In private interviews, the village women expressed a wide range of attitudes towards individual methods. Those women who approved of and/or used temporary methods often had more education, were of higher relative socioeconomic status, and attributed their acceptance of modern methods to increased exposure to knowledge. These women felt that there were several reasons to use spacing methods: in order for women to recover physically from labour before another pregnancy; to avoid the challenges of household duties with multiple infants to care for; and the need to provide a full course of breastfeeding to each.

An interviewee aged 33 years stated, “One baby is crying, the other one needs to be taken to school, I have to do the cooking and other household tasks … you need a three-year gap between children to keep things manageable.”

Five of 15 women interviewed individually had previously used modern reversible methods; however, none was currently using them (Table 1). Eight of the women had undergone female sterilization procedures. Several women had attempted to use contraceptive pills; all had stopped using these due to side-effects. Several older village men have had vasectomies; however, none of the younger men has undergone the procedure.

**Table 1:** Selected demographic characteristics of interviewees

Interviewee	Age (years)	Living children		Years of education	Modern contraceptive method use*		Past use of OCP to delay menstruation
		Sons	Daughters		Ever use	Current use	
1	36	2	0	12	IUD, FS	FS	Yes
2	45	2	1	5	FS	FS	Yes
3	33	0	2	10	IUD, FS	FS	Yes
4	25	0	1	8	None	None	Yes
5	33	3	0	9	FS	FS	No
6	40	1	3	0	FS	FS	Yes
7	30*	3	0	3	None	None	No
8	27	1	2	3	FS	FS	Yes
9	32*	1	1	9	None	None	Yes
10	35	1	1	0	None	None	Yes
11	27	1	0	7	OCP	None	Yes
12	28	0	2	10	OCP, IUD	None	No
13	28	0	2	7	Condoms, OCP	None	No
14	53	1	2	7	FS	FS	Yes
15	37	1	3	0	FS	FS	Yes

*Ages are approximate; FS=Female sterilization; IUD=Intrauterine device; OCP=Oral contraceptive pill

Women in focus groups initially stated that the village women did not use modern reversible contraception. When asked if they had ever heard of any village women using modern methods, they stated that some women used female sterilization and that a very few women ‘might have' used IUDs or condoms. Information on individual contraceptive use for women in focus groups was not obtained. However, Table 2 lists selected overall demographic characteristics of the groups.

**Table 2:** Demographic characteristics of focus groups

Focus group	Age range (years)	No. of participants	Socioeconomic status*	Mean number of children/woman
1	20–27	11	High	Unavailable
2	19–25	9	Medium	0.5
3	25–37	8	Low	2.25
4	27–50	11	Medium	3.45
5	23–46	9	Medium	1.7
6	28–49	14	Low	3.07

*Socioeconomic status compared to the rest of the village

A focus group 4 participant aged 50 years reported, “Most women in our hamlet do not use any contraceptive methods. Maybe some younger women might be able to answer your questions.”

“Interviewer: Do women here use family planning? (pause) A focus group 2 respondent aged 25 years: Yes, but only a few women. Another focus group 2 respondent aged 24 years: We know about the copper-T and pills, but no one here uses them.”

“Some women might use it, but they use it secretly; so, no one else knows”, stated a focus group 1 participant aged 27 years.

When women were asked in interviews about the discrepancy between the focus-group responses and their own accounts, they stated that women were likely uncomfortable with the subject of contraception for various reasons.

According to an interviewee aged 40 years, “Only educated people might use contraceptives—illiterate people do not even know about them, and if they do, they may be afraid of the side-effects since they have to work hard in their fields.”

“Interviewer: During the group discussion, the women at first would not admit that they know of or that they have used contraceptives; but later as we explained about the different methods, they opened up and discussed them with us. Why did that happen?

Interviewee: Women conceal the facts. They always do that when anybody asks us about it. They have to be told over and over again not to lie. They know all about contraception but they want to show that they have achieved spacing without using anything”—from discussion with an interviewee aged 36 years.

The village women often achieved their ideal birth intervals (36–60 months) and limits (most women desired two children). Exclusive breastfeeding was rare after the infant had reached six months of age, although supplemental nursing could continue for up to two years. When asked what methods they used for spacing and limiting children, women primarily cited ‘self control', a fascinating term with several meanings. In reference to sexual relations, the term is used in a general sense to refer to the Hindu virtue of moderation and self-discipline. However, in the village context, it also refers to the specific practices of abstinence and withdrawal, both of which are considered admirable exercises of restraint. The ability to control oneself sexually is regarded as an important personal virtue. Several women in focus groups commented (to general agreement) that parents of three or more children were irresponsible.

A focus group 2 participant aged 24 years stated, “If a family has two children, both children can get a good education. So, those who have two children or less are approved of by other people. If someone has more than five children, everyone knows they would not be able to educate them all.”

Self-control was often used as an adjunct to the ‘safe days' method of contraception, a form of periodic abstinence that many village women said that they used successfully. While most women in the area were aware that there were certain times during a woman's cycle when she was more likely to conceive, every woman in the sample who identified the ‘safe days' method as a means of contraception stated that, to avoid pregnancy, couples should abstain from intercourse during the first 5–10 days of the woman's menstrual cycle and the last 5–10 days. However, in physiological terms, this belief is entirely incorrect: the ‘safe' mid-cycle interval is, paradoxically, the time when a woman is most likely to conceive.

The success that women attribute to this method may be due to the fact that the likelihood of conceiving, even when using no method of contraception, is low when intercourse can only occur during short 2–3-week intervals several times a year: most village men migrate to larger cities for most of the year and return only for short periods during festivals. Also, extended breastfeeding likely provides limited protection against pregnancy. Women acknowledged that the economic migration of men was the primary reason that they did not use temporary methods of contraception. Most felt that they would use contraception if their husbands lived in the area. Women did not want to adopt methods that they believed must be continued even when their husbands were away working. Several women stated that using long-term contraception while their husbands were away might cast the socially-devastating suspicion of infidelity on them.

In addition to their perceived self-control, women's social prestige is highly influenced by literacy and educational level, although this may serve as a proxy for socioeconomic status. The need to provide for an adequate education for children—and, thus, by extension, improve the family's status—was frequently cited as a main motivation limiting family size.

An interviewee aged 32 years stated, “If we have only two children, we can afford to send them to school. If we have any more, we would not be able to care for them properly. If we cannot provide for any more, why have them and make them suffer?”

A focus group 3 participant aged 36 years said, “After two children, there is no use getting pregnant again. Children need proper education and clothing and so on. If a woman does not bother to use contraception, though, she may have any number of children.”

Abortion was used often as a back-up method of contraception when a woman conceived shortly after giving birth or the ‘days' method failed. Although abortion on demand is legal in India and available in the *taluka* capital, it is an expensive procedure, costing around Rs 1,500 (approximately US$ 36), equivalent to almost two months' wages for a woman working in the field. Abortion was seen as dangerous and shameful—the result of sexual immoderation—and women were forced to conceal it.

An interviewee aged 36 years said, “Nowadays, some women do not use any methods like the copper-T or pills, but then they go to get an abortion secretly if they get pregnant.”

Another interviewee aged 53 years stated, “If her first baby is only 5 or 6 months old and a woman gets pregnant again, she will get an abortion. But she keeps it secret and tells no one … women should not have abortions because the suffering is worse than labour. It is a sin.”

### Social risks: family pressures and expectation

Women often described an ideal of reproductive autonomy within the extended family that was at odds with the experiences they related of attempts to limit family size.

A focus group 1 participant aged 33 years said, “It is the responsibility of both partners to decide how many children to have because it is a matter between a husband and a wife.” “Our in-laws want children, but the husband and wife should make the final decision on how many children to have.”

Another focus group 4 participant aged 37 years stated, “Both husband and wife should make the decision. My husband and I were happy with two daughters but my mother-in-law wanted a grandson; otherwise, I would have gotten the operation. Instead, I have five children.”

Joint families consisting of a husband and a wife, their children, and the husband's parents, and often other relatives, are the norm in the village. Most women stated that the decision when to have children and when or if to continue bearing children should be made by the husband and wife together, with the wishes of other family members a secondary concern. The role of elder relatives living within the extended household in determining family size varied among the families of the study women. Several women bore one or more child(ren) beyond their desired number to appease their in-laws; a few found their in-laws to be supportive and encouraging in their use of contraception.

A focus group 5 participant aged 28 years said, “Mothers-in-law insist that you need a son to inherit and say that since they [the grandparents] are able to provide for more children, why not keep trying till you have a son?”

“My mother-in-law and father-in-law have said that our two are enough”, said a focus group 2 participant aged 24 years.

Some older relatives pressured couples to continue bearing children and forbid them to use contraception. The close proximity of extended family members sometimes prevented the use of contraception by women, even those with consenting husbands; this presented a dilemma for women who faced pressure at home to bear additional children but feared social ridicule in the village for doing so. The Hindu beliefs of the village included a strong menstrual taboo where women are viewed as ritually polluted during the first three days of their menstrual cycle, and women are prohibited from cooking, entering the kitchen, touching eating utensils, sleeping in the conjugal bed with their husbands, and engaging in religious activities. The monthly change in menstruating women's household routine allowed other household members to monitor her fertility patterns.

An interviewee aged 28 years stated, “My husband wants a son but I do not … the rest of the family would not allow us to get the operation. I can have it done secretly but they will figure it out quickly when I do not get pregnant again.”

### Side-effects

Copper-containing IUDs were the most commonly-used reversible method; however, the perception of increased bleeding and cramping associated with their use made women wary. They believed that the IUD damages the uterus and health (many mentioned the danger that an IUD would go ‘up inside the uterus'); because, the use of an IUD often causes heavier, longer and more frequent menses. Women who used IUDs faced exclusion from domestic and religious activities more frequently due to the prevailing menstrual taboo. Several women expressed the belief that IUDs require monthly check-ups, a prohibitive expense in terms of time spent away from the household and fields. Many women thought that failure to remove the copper-T promptly at three years would result in complications.

A focus group 3 participant aged 37 years reported, “My daughter was fitted with the copper-T. It went inside her uterus and then she got pregnant. When she had the baby, she had a lot of trouble with the delivery.”

“I know one lady who had a copper-T and forgot to take it out on the exact date. It got inside her womb and because of that, she had to have a hysterectomy”, said a focus group 4 participant aged 40 years.

The village women were aware of the existence of oral contraceptive pills; however, a few used them for contraception. Instead, they used them to circumvent the restrictions of the menstrual taboo by taking one or several pill(s) over a small number of days to delay menstruation. Most study women had taken pills for this reason. Most did so without guidance by a physician and may have been taking large doses of hormones, leading to an increased risk of side-effects. Women described symptoms of severe abdominal pain, nausea and vomiting, vertigo, and greatly increased vaginal discharge and infections far in excess of those normally found when oral contraceptives are used as directed by physicians.

A 53-year old interviewee said, “I took pills once for five days during the Ganpati Festival, but they caused a lot of pain. My husband cautioned me not to use them again because I was too sick to work while I was taking them.”

Another interviewee aged 25 years said, “Before I was married, I once took those pills for 2 or 3 days—the side-effects were heavy discharge and pain in my abdomen.”

### Vasectomy and female sterilization

Although female sterilization was the most prevalent method in the village, male sterilization was extremely uncommon, despite the availability of both operations for no cost in a nearby town easily reachable by bus (and while both men and women undergoing sterilization receive an incentive payment, the payment for vasectomy is greater, Rs 500 [US$ 12] vs Rs 1,500 [US$ 36]). Despite the larger amount of money awarded for vasectomy, most women felt that female sterilization is a better option for their families, regardless of its more invasive nature and any willingness on the part of husbands to undergo the procedure.

Rice and fruit farming are the primary income-generating activities in the village, involving lifting, bending, and tree climbing. The study women believed that vasectomy procedures often limited the ability of a man to climb, lift, and stoop, permanently disabling men, thus removing the primary source of income of the household. Economic contributions of men were more highly valued than any of women's contributions to the household: despite the fact that women believed that female sterilization causes back and abdominal pain and could disable them, they felt that it was better for the woman to be debilitated than the man.

A focus group 3 participant aged 33 years stated, “The men who had vasectomies a long time ago are still suffering. They feel pain in their hips and lower backs when they try to climb trees. Men who have office jobs can risk a vasectomy, but men who work on farms cannot risk the pain because they have to be able to climb trees.”

A 45-year old interviewee said, “I would not let my husband get a vasectomy. Female sterilization is better … men have to labour the whole day, and why should they (men) suffer for us when they are earning for the family.”

A focus group 4 participant aged 36 years said, “If a woman gets sick after her operation, her husband can manage the household. But if her husband, who earns a higher wage, cannot work after he gets the operation, they cannot run the household with the 30 rupees she earns per day.”

Desire for economic stability provided a motivation for sterilization: if a woman felt that her husband might not be able to provide financial support due to poor health or alcoholism, or that additional pregnancies and births would disable her, she might feel that it was in her best interest and that of her existing children to limit her fertility. The village women also believed that vasectomy damaged sexual potency and overall strength of men.

A focus group 3 participant aged 33 years said, “If I had a civil service job with a fixed salary, say, I could support my household. But with just 25 rupees a day, how could I? Those 5 or 6 men who had vasectomies are sitting at home and not earning much.”

A 28-year old interviewee reported, “My mother has told me stories [about female sterilization] about big machines and huge scissors, that scare me … but we cannot let men get vasectomies, because they suffer more and cannot climb trees afterwards, so I will probably get the operation anyway.”

The majority of the vasectomized men in the vill-age underwent the procedures during mass sterilization campaigns of the 1970s, where informed consent was frequently non-existent. These men attributed various illnesses and weakness to the procedure. Misconceptions about the mechanics of vasectomy—that part of the penis is removed, that a main nerve is severed, and that a major artery is cut—contributed to women's disapproval of the operation and might have influenced men's self-perception of disability. The potential of vasectomy failure was a strong disincentive for use because of the doubt such failures cast on the fidelity of a wife.

“After hearing about all the problems, the men who had experiences of vasectomies, no wife would allow her husband to get one”, stated a focus group 6 participant aged 27 years.

A 33-year old interviewee said, “Sterilization is better because vasectomy fails sometimes, and if a woman then gets pregnant, her husband will think that she was unfaithful.”

Several women disagreed that female sterilization was a better method than vasectomy, but none of them believed that village men would find vasectomy acceptable, despite what they perceived as its advantages, nor did they feel that economic incentives would address the problem.

A 32-year old interviewee stated, “Men pressure their wives for the operation. I asked my husband once to get a vasectomy, but he said he could not. He cannot even handle a single injection; the operation would be impossible. He told me, ‘You are brave, you get it done instead'.”

Another interviewee aged 53 years said, “The men get paid to get vasectomies; so, if they get it done, that is the only reason. Then they waste all the money on alcohol—they just want the money for drinking.”

### Lack of information

The study women had varying levels of knowledge of contraception. Their primary source of information was female relatives and friends, although educated women had some exposure in school. Women in several hamlets stated that family-planning officials had never visited them while others had had negative experiences with auxiliary nurse-midwives (ANMs). Several focus-group and interview participants were nursery school teachers who were instructed to ‘get cases', i.e. to find village women who wanted sterilization and act as a liaison with the local hospitals.

“One of the old ANMs was rude and just wanted to get cases. She used to bark like a dog at every lady who had a baby and insist that they get the operation without explaining it to them”, reported a focus group 4 participant aged 40 years.

“We have only been given information about steri-lization. We only talk to women who are interested and register them for the operation. No information about pills or condoms, etc. is given to us at all”, reported a focus group 2 participant and nursery teacher aged 25 years, whose job includes ‘getting cases'.

The study women expressed a wish to learn more about family-planning methods—both for themselves and for their daughters. Many women stated that the only method that they were given information about was sterilization and that they knew little about other methods. Although such information is available through doctors, many women cannot afford the time and money to travel to a clinic for what they view as non-essential healthcare.

A 40-year old interviewee reported, “Dr. [name] comes for sterilization but he does not tell us anything about contraceptives.”

Another 32-year old interviewee stated, “The government health workers give out correct information, but women do not trust it if they do not already know the worker. I am comfortable answering your questions because I know you [speaking to the translator] so well.”

## DISCUSSION

Although a relatively few study women used modern reversible methods of contraception, the prevalence among interviewees was far higher than it would have been based on the information from focus groups. ‘Self-control', i.e. sexual moderation (defined in the village as the use of withdrawal or periodic abstinence), was valued extremely highly by the village women. In focus groups, the women stated that contraception was often unnecessary since couples were expected to practise self-control, and they expressed pity for those women whose husbands would not cooperate. However, in private in-depth interviews, the women revealed the widespread use of modern methods, particularly female sterilization with limited use of other methods, and abortion: ‘self-control' was not totally effective. Unplanned pregnancies still occurred. When a woman found herself pregnant with an unwanted child, she either bore it (often to appease older relatives) or used abortion, which women viewed as expensive, dangerous, regrettable, shameful, and physically taxing, but necessary. Using self-control may be a way of negotiating fertility decisions without having to violate social norms of modesty and restraint with open discussion.

We theorize that demand for modern contraceptive methods is limited primarily by stigma surrounding what women perceive as their (or their husband's) lack of sexual self-regulation. Although the village women stated that female sterilization was not necessary if a couple could practise self-control, it was a much more accepted procedure. This may be due to the fact that couples with more than three children were perceived to be sexually immoderate and are often teased and commented upon in the village. Closely-spaced births, while considered unwise, did not elicit the same public social condemnation as large families did.

Demand was further limited by the ability of many women to achieve their fertility goals without the use of modern reversible methods. Mistaken beliefs regarding contraceptive mechanisms, particularly vasectomy, and fear of side-effects, especially increased vaginal bleeding and discharge, also prevented women from seeking out modern reversi-ble methods. Women associated physical and social risks with the use of temporary methods and viewed female sterilization as superior to temporary methods. Vasectomy was considered disabling and economically foolhardy.

Contraceptive information in the village was passed from woman to woman. Women had mistaken beliefs about the mechanisms and side-effects of reversible female methods and vasectomy—that IUDs need monthly check-ups from the healthcare provider, that they cause extensive damage to the female reproductive tract if not removed promptly, and that they cause vaginal and uterine prolapse; that vasectomies are performed by removing part of the penis, severing a nerve, or cutting a blood vessel.

In this context, women communicated their experiences of frightening and inconvenient side-effects with little anatomical or physiological knowledge to understand what was happening to their bodies. Most of these side-effects, such as nausea, dizziness, increased vaginal discharge, and weight gain with oral contraceptive pills, and increased bleeding and cramping with IUDs, are well-documented in the medical literature. However, for the study women who had used these methods or who have heard other women speak of their experiences, such events took place with little or no understanding of the physiological underpinnings and caused great fear and apprehension. The belief that oral contraceptive pills cause severe vertigo, stomach pain, and nausea may be associated with the practice of taking large doses of pills for brief periods to delay menses.

Familial and social expectations, in addition to those surrounding ‘self-control', played a large role in women's fertility and contraceptive decisions. Son preference, although not as strongly expressed in this area as in some others in India (24-27), is present in the village. Women faced a dilemma: they expressed that they felt expected to provide sons, but women who bore multiple daughters in an attempt to bear males were regarded as lacking sexual restraint. The strong disapproval that the study women felt for vasectomy demonstrates women's perception of the central economic role of men's wages to households. Many women also worked in the fields but for much smaller wages. The non-wage labour that women contribute to the household was considered to be of lesser value than any men's work. The fear of loss of male economic contribution perpetuated reliance on female sterilization.

In the study village, access to contraceptive services and supplies was not a primary limiting factor in women's use of reversible modern methods. A range of temporary family-planning methods was available free of charge at health posts and at nominal cost at pharmacies, although the lack of public transportation, time, and privacy likely still limit willingness of women to access these resources. However, when women felt that they had no other options, they found ways to obtain medical termination of pregnancy and female sterilization services in the *taluka* capital.

Lack of knowledge also limited demand. The women demonstrated poor knowledge of temporary methods. The coverage of the village by the health workers was inconsistent. Most villagers did not own a radio or a television, and access to media was limited for all but the wealthiest families; media access has positively correlated with contraceptive use in India, as has education of women (28). Women's lack of correct knowledge regarding mechanisms and side-effects directly affected the risks that they perceived in using the temporary methods they know about. These perceptions were largely formed by the experiences other women related to them, which, in turn, were coloured by misunderstandings. This social learning process may offer a way to educate women about contraception: by training other women in the community to teach them. Those women who had lived in urban areas, who were literate, and who had attended secondary school were often looked up to by other village women as a source of contraceptive and general knowledge, and such women could potentially be used in the dissemination of family-planning information to the village women. However, provision of correct information may not substantially increase the uptake of modern methods, given the social barriers to using them, or even admitting the need for them.

The primary limitations of this study were the small sample size, particularly of women who participated in in-depth interviews, the inclusion of a single village, and the potential for bias introduced by using a snowball sampling method (with self-selection into focus groups). The sample size was limited by the seasonality of the study period: the study took place during the three weeks prior to and the month immediately following the onset of monsoon rains. As farming is the major economic activity of the village and women actively participate (due in part to the dearth of able adult males), most women were occupied in these tasks and did not have time to participate. Since the focus groups were held first, prior to the beginning of the rains, more women were able to attend. Because we sought to capture the perspectives of women of varying socioeconomic levels, we did not limit our interview sample to those women who did not work in the fields. While this limited the number of women with whom we spoke, we believe that the conclusions of our study provide a more complete description of the perspectives of all the village women. Additionally, after both the six focus groups and 15 interviews had been conducted, we found that we were approaching information saturation in each section.

We feel that our small-scale approach, where we approached women with the assistance of a trusted local interpreter, provided us with a uniquely intimate perspective into women's beliefs surrounding this sensitive (and in our findings, stigmatized) topic.

Social norms of sexual self-control and modesty, family pressures, and a set of myths and misconceptions regarding reversible methods of contraception limit demand for modern reversible methods of contraception, even among women who clearly express an unmet need. The low use of reversible contraceptive methods was also driven by women's belief that modern reversible methods were not only unnecessary but also risky to health and social position within the family and the village as a whole. Although these results suggest several possible policy interventions, the strong stigma surrounding modern reversible methods of contraception may only be remedied by time, as the social norms of the village catch up to the two- or three-child ideal espoused by the study women.

Community-based outreach programmes that facilitate public discussion of family planning may help de-stigmatize admission of unmet need to contraceptive providers. Women rely on other women within their social network for information regarding health in general and family planning in particular. It is, therefore, of vital importance that those women who do choose to use reversible methods are counselled before and during contraceptive use. Failure to do so may result not only in discontinuation on the part of the user, but also in a domino effect preventing other women with unmet need from using contraception.

Women must be educated on both their own anatomy and physiological processes, particularly the menstrual cycle and effective periodic abstinence, and the mechanisms of different methods so that they can understand the side-effects they experience and make an informed decision regarding methods. However, many women will likely choose to continue using traditional methods, as they are satisfied with the results they can achieve, and consider the use of abortion as a back-up method to be an acceptable risk (14). Educational interventions can focus on increasing demand by educating women on specific contraceptive methods to reduce the role of myths and misperceptions as barriers. Within the village, those women who have some secondary education have increased social capital; workshops and trainings that educate these women about family planning can lead to the dissemination of knowledge via a source that women trust.
